# Supplementary material for: The Systems Biology Research Tool: evolvable open-source software
Source: BMC Syst Biol. 2008 Jun 29;2:55. doi: 10.1186/1752-0509-2-55 (PMC2446383; doi:10.1186/1752-0509-2-55)
Supplement: Additional file 1 — SBRT Archive. An archive of the current version of the Systems Biology Research Tool. [file 1752-0509-2-55-S1.zip › sbrt-1.4.0/doc/developers_guide/api/sbrt/shell/text/math/SenseFormat.html]

SenseFormat


|  |  |  |
| --- | --- | --- |
| **Overview** \| **Package** \| **Class** \| **Use** \| **Tree** \| **Deprecated** \| **Index** \| **Help** | |  |
| **PREV CLASS**   NEXT CLASS | **FRAMES**    **NO FRAMES**     **All Classes** |  |
| SUMMARY: NESTED \| FIELD \| CONSTR \| METHOD | DETAIL: FIELD \| CONSTR \| METHOD |  |


---


## sbrt.shell.text.math Class SenseFormat

```
java.lang.Object
  sbrt.shell.text.math.SenseFormat
```

**All Implemented Interfaces:**: Format, Formatter<OptimizationSense>, Parser<OptimizationSense>, SimpleFormat<OptimizationSense>

---

``` public class SenseFormat extends java.lang.Object implements SimpleFormat<OptimizationSense> ```

This class is used to represent formats of optimization senses.
The strings `"Maximize"` and `"Minimize"` are used to
represent optimization senses.

**Author:**
:   This class was written and documented by Jeremiah Wright
    while in the Wagner lab.

---

**Constructor Summary**

`SenseFormat()`


| **Method Summary** | |
| --- | --- |
| `<S extends OptimizationSense> java.lang.String` | `format(S obj)`             Returns a formatted string representation of the provided optimization sense. |
| `OptimizationSense` | `parse(java.lang.String source)`             Parses the provided string and returns its corresponding object. |

**Methods inherited from class java.lang.Object**

`clone, equals, finalize, getClass, hashCode, notify, notifyAll, toString, wait, wait, wait`

**Constructor Detail**

### SenseFormat

```
public SenseFormat()
```


**Method Detail**

### format

```
public <S extends OptimizationSense> java.lang.String format(S obj)
```

:   Returns a formatted string representation of the
    provided optimization sense.

    :   **Specified by:**: `format` in interface `Formatter<OptimizationSense>`
    :   **Type Parameters:**: `S` - a type that extends the primary type of this format. **Parameters:**: `obj` - the optimization sense to be formatted. **Returns:**: a formatted string representation of the provided optimization sense.

---


### parse

```
public OptimizationSense parse(java.lang.String source)
```

:   Parses the provided string and returns its
    corresponding object.

    :   **Specified by:**: `parse` in interface `Parser<OptimizationSense>`
    :   **Parameters:**: `source` - the string to be parsed. **Returns:**: the object corresponding to the provided string.


---


|  |  |  |
| --- | --- | --- |
| **Overview** \| **Package** \| **Class** \| **Use** \| **Tree** \| **Deprecated** \| **Index** \| **Help** | |  |
| **PREV CLASS**   NEXT CLASS | **FRAMES**    **NO FRAMES**     **All Classes** |  |
| SUMMARY: NESTED \| FIELD \| CONSTR \| METHOD | DETAIL: FIELD \| CONSTR \| METHOD |  |


---
